# Immune-Related Adverse Events in a Patient With Burkitt's Lymphoma During PD-1 Immune Consolidation Therapy: A Case Report

**DOI:** 10.1155/crh/9967011

**Published:** 2025-10-29

**Authors:** Qi Zhang, Fankai Meng, Yang Cao, Xiaojian Zhu, Yicheng Zhang, Yi Xiao

**Affiliations:** Department of Hematology, Tongji Hospital, Tongji Medical College, Huazhong University of Science and Technology, Wuhan, Hubei, China

**Keywords:** Burkitt's lymphoma, case report, immune-related adverse events, PD-1

## Abstract

A patient with Burkitt's lymphoma developed upper gastrointestinal adverse events during immune consolidation therapy following chemotherapy combined with autologous stem cell transplantation. Subsequent administration of vedolizumab as adjunctive therapy resulted in improvement of the patient's symptoms. This case is expected to provide a reference for the expanded application of PD-1 inhibitors in non-Hodgkin's lymphoma.

## 1. Introduction

Programmed cell death protein 1 (PD-1) and programmed cell death-ligand 1 (PD-L1) are highly expressed immune regulatory molecules in lymphomas. Antibodies targeting PD-1 or PD-L1 can reduce T-cell exhaustion and restore the immune response against cancer cells [1]. Research on α-PD-1/PD-L1 inhibitors combined with chemotherapy, radiotherapy, angiogenesis inhibitors, targeted therapy, and other immune checkpoint inhibitors (ICIs) is currently underway, showing excellent antitumor efficiency and a high response rate [2]. It has been found that nivolumab, a PD-1 inhibitor, can enhance the cytotoxic activity of cytarabine and ifosfamide on Raji cells in the second-line chemotherapy regimen [3]; thus, it is essential to pay attention to its safety. Gastrointestinal (GI)-irAEs are currently one of the most common toxicities in ICIs, early and accurate diagnosis, and appropriate treatments are critical [4]. Here, we report a case of Burkitt's lymphoma in which an upper GI adverse event occurred during immune consolidation therapy after chemotherapy combined with autologous stem cell transplantation, expected to provide a reference for the broader application of PD-1 inhibitors in non-Hodgkin's lymphoma.

## 2. Case Presentation

**Figure 1:**
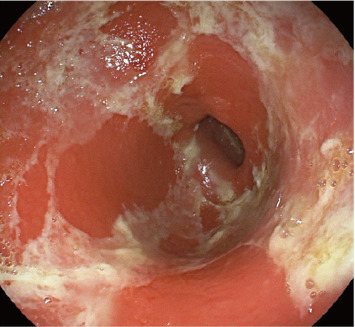
Gastroscopic images.

**Figure 2:**
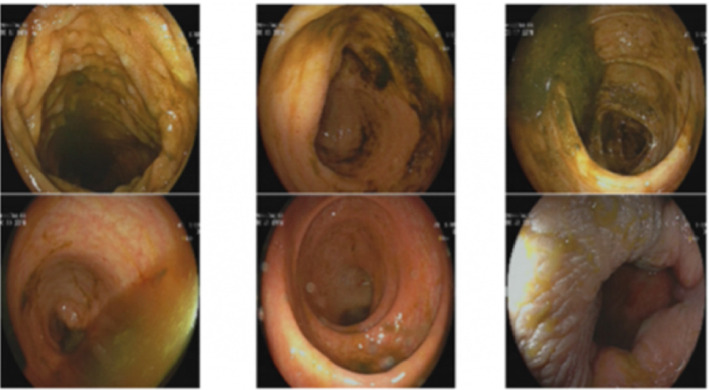
Videography results of cases. Electronic colonoscopy showed no organic lesions in the whole large intestine.

An 18-year-old patient underwent an appendectomy at a local hospital in September 2020 due to pain in right lower abdominal quadrant. Positron emission tomography–computed tomography revealed thickened intestinal wall of the ileum terminal and ileocecum, multiple enlarged lymph node shadows in the bilateral internal mammary and diaphragm regions, unevenly sunken and diffusely thickened right pleura, falciform ligament, omentum sac, omentum, mesentery, pelvic cavity, and enhanced metabolism. Peritoneal and pelvic effusions (partially wrapped) were also observed, considered multiple infiltrations. Pathology revealed non-Hodgkin's lymphoma, probably Burkitt's lymphoma. Immunohistochemical staining showed the following: CK (−), CD20 (+), CD5 (−), Desmin (−), and Ki67: about 99% (+). The patient presented to our hospital in October 2020. Next-generation sequencing identified mutations in *RHOA, DDX3X, SMARCA4, IGLL5, PTEN, SETD2, DNMT3A, NOTCHI, PLCGI, KMT2D,* and *MAP2KI* genes. From November 2020 to February 2021, the patient received multiple chemotherapies, including the hyper-CVAD-A regimen (cyclophosphamide, vincristine, doxorubicin, and dexamethasone) and the hyper-CVAD-B regimen (methotrexate and cytarabine). During this period, 600 mg rituximab were administered to the patient. The patient received a transplant at our hospital on May 14, 2021. Later, she received regular 200 mg PD-1 inhibitors treatment in August 2021. In February 2022, the patient's appetite began to decrease and gradually worsened, accompanied by nausea and vomiting. She was diagnosed with “gastroenteritis” in our hospital and recovered after receiving organ protection and nutritional supplements. However, the patient later presented with symptoms including loss of appetite, vomiting, dull epigastric pain, belching, and acid reflux. Gastroscopy at a local hospital revealed pyloric stenosis (Figure 1). The patient received nutritional support and organ-protective antiulcer therapy comprising proton-pump inhibitors and gastric mucosal protectants. A jejunal feeding tube was placed; yet the patient's symptoms showed no improvement. After transfer to our hospital, electronic colonoscopy was performed to further evaluate the GI tract, which showed no organic lesions in the entire large intestine (Figure 2). The patient exhibited a poor response to glucocorticoid therapy and was subsequently treated with intravenous methylprednisolone in combination with ruxolitinib (5 mg bid from April 15–24) and rituximab (200 mg qd from April 15–17), this regimen provided no symptomatic relief. Given the patient's immune-related GI issues, vedolizumab therapy was initiated. The patient received intravenous vedolizumab (300 mg) on April 19 and 23. Concurrent medications included intravenous methylprednisolone (10 mg) and oral budesonide (3 mg) from April 19 to 24. Remarkably, this combination therapy gradually relieved the patient's pyloric stenosis symptoms, leading to discharge. After a 1-year telephone follow-up, we confirmed that the patient remained in good condition, and patient's weight recovered to approximately 48 kg.

## 3. Conclusion and Discussion

PD-1 inhibitors have proven very effective in treating relapsed and refractory malignant lymphoma; however, it can also cause immune-related adverse effects, there have been reports of PD-1 inhibitor-induced acute erosive hemorrhagic gastritis and gastric stenosis [5, 6]. In our case, regular PD-1 inhibitors therapy was administered after chemotherapy and autologous stem cell transplantation to achieve immune consolidation effects. However, the patient developed pyloric stenosis after receiving PD-1 inhibitors, resulting in loss of appetite and vomiting after eating (although the patient was yet to undergo gastroscopy at our hospital due to an indwelling jejunal feeding tube in the external hospital). After immune-related adverse events diagnosis, definite causes such as infection should be excluded. The patient had no history of peptic ulcers or tumor-related diseases in the past (especially before the use of PD-1 inhibitors). This confirmed our diagnosis to a certain extent.

The patient in this study did not respond well to glucocorticoid treatment; this observed heterogeneity may arise from interindividual variability or organ-specific factors. To expeditiously alleviate the patient's symptoms, combination therapy with glucocorticoids, ruxolitinib, and rituximab was administered but failed to alleviate symptoms. Given the immune-mediated etiology of the patient's GI involvement, vedolizumab was initiated. Combined with adjunctive therapies, this intervention achieved progressive resolution of pyloric stenosis symptoms, and a small amount of liquid food could be consumed.

Vedolizumab, a known intestinal-selective monoclonal antibody against α4β7 integrin, has been approved to treat ulcerative colitis and Crohn's disease in moderate to severe active stages [7]; a study found that vedolizumab is effective against ICI-induced colitis [8, 9]. Notably, a randomized, double-blind, Phase 3 trial demonstrated the superiority of vedolizumab (an anti-α4β7 integrin monoclonal antibody) over placebo in preventing acute graft-versus-host disease (aGvHD) affecting the lower GI tract following allogeneic hematopoietic stem cell transplantation [10]. Given its established efficacy in inflammatory bowel disease (IBD) and other inflammatory conditions [9, 11], vedolizumab has emerged as a potential therapeutic option for steroid-refractory/resistant (SR) GI aGvHD. A key advantage of vedolizumab lies in its gut-selective mechanism of action, which localizes immunosuppressive effects to the GI tract, thereby minimizing systemic exposure [12].

In conclusion, we report an immune-related adverse event in a patient with Burkitt's lymphoma after receiving PD-1 immune consolidation therapy. The diagnosis and treatment of this case may provide some guidance for the wide use of PD-1 in the future. However, the specific underlying mechanism remains unclear. Moreover, the patient had symptoms of vomiting and an inability to eat due to pyloric stenosis, but the colonoscopy showed no lesions in the whole colon. Why the more common lower digestive tract immune-related adverse events did not appear in the patients in this study, further analyses are required.

## References

[1] Sgambato A., Casaluce F., Sacco P. C. (2016). Anti PD-1 and PDL-1 Immunotherapy in the Treatment of Advanced Non-Small Cell Lung Cancer (NSCLC): A Review on Toxicity Profile and Its Management. *Current Drug Safety*.

[2] Yi M., Zheng X., Niu M., Zhu S., Ge H., Wu K. (2022). Combination Strategies With PD-1/PD-L1 Blockade: Current Advances and Future Directions. *Molecular Cancer*.

[3] Zhang R., Li X., Li Z. (2020). Study on Enhancing the Killing Activity of Chemotherapy Drugs by PD-1 Inhibitor on Raji Cell Line. *Zhonghua Xue Ye Xue Za Zhi*.

[4] Zhang M. L., Neyaz A., Patil D., Chen J., Dougan M., Deshpande V. (2020). Immune-Related Adverse Events in the Gastrointestinal Tract: Diagnostic Utility of Upper Gastrointestinal Biopsies. *Histopathology*.

[5] Ai Q., Chen W., Li Y., Li G. (2022). Upper Gastrointestinal Tract Iraes: A Case Report About Sintilimab-Induced Acute Erosive Hemorrhagic Gastritis. *Frontiers in Immunology*.

[6] Song K., Dong H., Jiang S. (2023). Case Report: A Rare Case of Sintilimab-Induced Gastric Stenosis and Literature Review. *Frontiers in Oncology*.

[7] Loftus E. V., Feagan B. G., Panaccione R. (2020). Long-Term Safety of Vedolizumab for Inflammatory Bowel Disease. *Alimentary Pharmacology & Therapeutics*.

[8] Nielsen D. L., Juhl C. B., Chen I. M., Kellermann L., Nielsen O. H. (2022). Immune Checkpoint Inhibitor-Induced Diarrhea and Colitis: Incidence and Management: A Systematic Review and Meta-Analysis. *Cancer Treatment Reviews*.

[9] Bergqvist V., Hertervig E., Gedeon P. (2017). Vedolizumab Treatment for Immune Checkpoint Inhibitor-Induced Enterocolitis. *Cancer Immunology Immunotherapy*.

[10] Chen Y. B., Mohty M., Zeiser R. (2024). Vedolizumab for the Prevention of Intestinal Acute GVHD After Allogeneic Hematopoietic Stem Cell Transplantation: A Randomized Phase 3 Trial. *Nature Medicine*.

[11] Boland B. S., Riedl M. A., Valasek M. A., Crowe S. E., Sandborn W. J. (2017). Vedolizumab in Patients With Common Variable Immune Deficiency and Gut Inflammation. *American Journal of Gastroenterology*.

[12] Ramos-Casals M., Brahmer J. R., Callahan M. K. (2020). Immune-Related Adverse Events of Checkpoint Inhibitors. *Nature Reviews Disease Primers*.

